# 7-Meth­oxy-3,4-dihydro­naphthalen-1(2*H*)-one

**DOI:** 10.1107/S1600536811020174

**Published:** 2011-06-11

**Authors:** Jerry P. Jasinski, James A. Golen, S. Sekar, H. S. Yathirajan, Nagaraja Naik

**Affiliations:** a Department of Chemistry, Keene State College, 229 Main Street, Keene, NH 03435-2001, USA; b Department of Studies in Chemistry, University of Mysore, Manasagangotri, Mysore 570 006, India

## Abstract

In the title compound, C_11_H_12_O_2_, the six-membered ketone ring fused to the 7-meth­oxy benzene ring adopts a slightly distorted envelope configuration with the central methyl­ene C atom being the flap. The crystal packing is stabilized by weak inter­molecular C—H⋯O and C—H⋯π inter­actions, which lead to supra­molecular layers in the *bc* plane.

## Related literature

For the synthesis of steroid estrogens, see: Belov *et al.* (2007 ▶). For the manufacture of important anti­depressant drugs, see: Shum *et al.* (2000 ▶). For multi-functional scaffolds of tetra­lone, see: Mahapatra *et al.* (2008 ▶). For related structures, see: Barcon *et al.* (2001 ▶); Haddad (1986 ▶); Orlov *et al.* (1996 ▶). For puckering parameters, see: Cremer & Pople (1975 ▶).
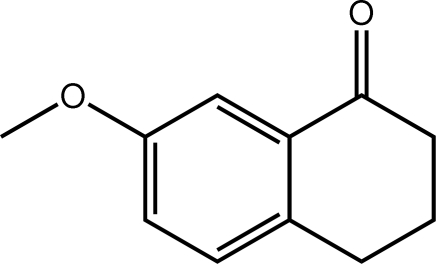


## Experimental

### 

#### Crystal data

C_11_H_12_O_2_
*M* *_r_* = 176.21
Monoclinic, 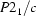

*a* = 7.4303 (4) Å
*b* = 7.4614 (4) Å
*c* = 16.4393 (8) Å
β = 90.976 (4)°
*V* = 911.27 (8) Å^3^
*Z* = 4
Mo *K*α radiation
μ = 0.09 mm^−1^
*T* = 170 K
0.35 × 0.25 × 0.10 mm

#### Data collection

Oxford Diffraction Xcalibur Eos Gemini diffractometer
Absorption correction: multi-scan (*CrysAlis RED*; Oxford Diffraction, 2010 ▶) *T*_min_ = 0.970, *T*_max_ = 0.991
8750 measured reflections
2345 independent reflections
1959 reflections with *I* > 2σ(*I*)
*R*_int_ = 0.023

#### Refinement

*R*[*F* ^2^ > 2σ(*F* ^2^)] = 0.044
*wR*(*F* ^2^) = 0.125
*S* = 1.04
2345 reflections
120 parameters
H-atom parameters constrained
Δρ_max_ = 0.26 e Å^−3^
Δρ_min_ = −0.17 e Å^−3^

### 

Data collection: *CrysAlis PRO* (Oxford Diffraction, 2010 ▶); cell refinement: *CrysAlis PRO*; data reduction: *CrysAlis RED* (Oxford Diffraction, 2010 ▶); program(s) used to solve structure: *SHELXS97* (Sheldrick, 2008 ▶); program(s) used to refine structure: *SHELXL97* (Sheldrick, 2008 ▶); molecular graphics: *SHELXTL* (Sheldrick, 2008 ▶); software used to prepare material for publication: *SHELXTL*.

## Supplementary Material

Crystal structure: contains datablock(s) global, I. DOI: 10.1107/S1600536811020174/tk2750sup1.cif

Structure factors: contains datablock(s) I. DOI: 10.1107/S1600536811020174/tk2750Isup2.hkl

Supplementary material file. DOI: 10.1107/S1600536811020174/tk2750Isup3.cml

Additional supplementary materials:  crystallographic information; 3D view; checkCIF report

## Figures and Tables

**Table 1:** Hydrogen-bond geometry (Å, °) *Cg*1 is the centroid of the C1–C3,C8–C10 ring.

*D*—H⋯*A*	*D*—H	H⋯*A*	*D*⋯*A*	*D*—H⋯*A*
C11—H11*C*⋯O1^i^	0.98	2.38	3.3095 (18)	157
C5—H5*A*⋯*Cg*1^ii^	0.99	2.77	3.6730 (14)	152

Symmetry codes: (i) 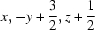; (ii) 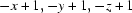.

**Fig. 1.**
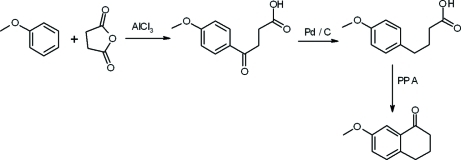
Reaction scheme for the title compound.

**Fig. 2.**
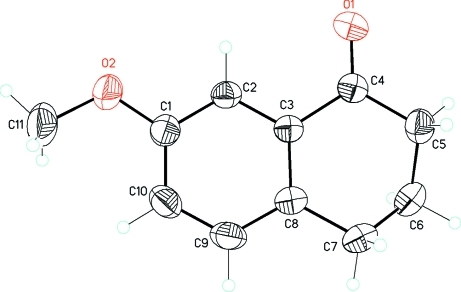
Molecular structure of the title compound showing the atom labeling scheme and 50% probability displacement ellipsoids.

## supplementary crystallographic information

### Comment

The structural and therapeutic diversity of small heterocyclic molecules continue to attract the attention of organic and medicinal chemists. Tetralones are emerging prominently as pharmacologically important bioactive molecules. Tetralone is an important and common intermediate in organic synthesis and is a ketone derivative of tetralin. The title compound (Systematic name: 3 ,4-Dihydro-7-methoxy-2(1H)-naphthalenone), (I), C_11_H_12_O_2_, is used in the preparation of agomelatine, which is an antidepressant. The importance of tetralone and its substituted derivatives as building blocks in the synthesis of steroid estrogens via isothiuronium salts is reported (Belov *et al.*, 2007). Tetralones are also important intermediates for the manufacturing of various serotonin inhibitor compounds having antidepressant activity, particularly sertraline, an important antidepressant drug (Shum *et al.*, 2000). Multi functional scaffolds of tetralone to generate further diversity with different functionalities is reported (Mahapatra *et al.*, 2008). The crystal structures of some related compounds, viz., 2,2-dibromo-3,4-dihydro-1(2H)-naphthalenone (Haddad *et al.*, 1986), 2-(4-nitrobenzylidene)-1-tetralone (Orlov *et al.*, 1996), (±)-1-tetralone-3-carboxylic acid and (±)-1-tetralone-2-acetic acid (Barcon *et al.*, 2001),have been reported. In view of the importance of tetralones, this paper reports the crystal structure of the title compound, (I).

In the title compound, C_11_H_12_O_2_, the six-membered ketone ring fused to the benzene ring adopts a slightly distorted envelope configuration (Cremer & Pople, 1975) with puckering parameters Q, θ and φ of 0.4869 (14) Å, 56.33 (15) ° and 185.10 (18) °, respectively (Fig. 1). For an ideal envelope θ and φ have values of 54.7° and 180°. Crystal packing is stabilized by weak C—H···O and C—H···π (Table 1) intermolecular interactions.

### Experimental

Anisole (4.3 ml, 0.040 mol) is acylated with succinic anhydride (4.2 g, 0.042 mol) in the presence of anhydrous aluminium chloride and nitrobenzene as solvent to give the intermediate keto acid. The keto group is reduced by hydrogenation with Pd/C as catalyst at 2-3 kgs pressure and 343-348 K for 2 -3 hours. Further work up, isolation and cyclization with poly phosphoric acid (PPA) gives 7-methoxy-1-tetralone (Fig. 1). X-ray quality crystals of (I) were obtained by slow evaporation from isopropyl alcohol (*M*.pt.: 333-336 K).

### Refinement

All of the H atoms were placed in their calculated positions and then refined using the riding model with Atom—H lengths of 0.95 Å (CH), 0.99 Å (CH_2_) or 0.98 Å (CH_3_). Isotropic displacement parameters for these atoms were set to 1.19-1.20 (CH, CH_2_) or 1.49 (CH_3_) times *U*_eq_ of the parent atom.

### Crystal data

**Table:**

C_11_H_12_O_2_	*F*(000) = 376
*M**_r_* = 176.21	*D*_x_ = 1.284 Mg m^−^^3^
Monoclinic, *P*2_1_/*c*	Mo *K*α radiation, λ = 0.71073 Å
Hall symbol: -P 2ybc	Cell parameters from 4312 reflections
*a* = 7.4303 (4) Å	θ = 3.7–32.3°
*b* = 7.4614 (4) Å	µ = 0.09 mm^−^^1^
*c* = 16.4393 (8) Å	*T* = 170 K
β = 90.976 (4)°	Block, colorless
*V* = 911.27 (8) Å^3^	0.35 × 0.25 × 0.10 mm
*Z* = 4	

### Data collection

**Table:**

Oxford Diffraction Xcalibur Eos Gemini diffractometer	2345 independent reflections
Radiation source: Enhance (Mo) X-ray Source	1959 reflections with *I* > 2σ(*I*)
graphite	*R*_int_ = 0.023
Detector resolution: 16.1500 pixels mm^-1^	θ_max_ = 28.7°, θ_min_ = 3.7°
ω scans	*h* = −9→10
Absorption correction: multi-scan (*CrysAlis RED*; Oxford Diffraction, 2010)	*k* = −9→10
*T*_min_ = 0.970, *T*_max_ = 0.991	*l* = −21→22
8750 measured reflections	

### Refinement

**Table:**

Refinement on *F*^2^	Secondary atom site location: difference Fourier map
Least-squares matrix: full	Hydrogen site location: inferred from neighbouring sites
*R*[*F*^2^ > 2σ(*F*^2^)] = 0.044	H-atom parameters constrained
*wR*(*F*^2^) = 0.125	*w* = 1/[σ^2^(*F*_o_^2^) + (0.0642*P*)^2^ + 0.1602*P*] where *P* = (*F*_o_^2^ + 2*F*_c_^2^)/3
*S* = 1.04	(Δ/σ)_max_ < 0.001
2345 reflections	Δρ_max_ = 0.26 e Å^−^^3^
120 parameters	Δρ_min_ = −0.17 e Å^−^^3^
0 restraints	Extinction correction: *SHELXL97* (Sheldrick, 2008), Fc^*^=kFc[1+0.001xFc^2^λ^3^/sin(2θ)]^-1/4^
Primary atom site location: structure-invariant direct methods	Extinction coefficient: 0.053 (7)

### Special details

**Table:**

Geometry. All esds (except the esd in the dihedral angle between two l.s. planes) are estimated using the full covariance matrix. The cell esds are taken into account individually in the estimation of esds in distances, angles and torsion angles; correlations between esds in cell parameters are only used when they are defined by crystal symmetry. An approximate (isotropic) treatment of cell esds is used for estimating esds involving l.s. planes.
Refinement. Refinement of *F*^2^ against ALL reflections. The weighted *R*-factor *wR* and goodness of fit *S* are based on *F*^2^, conventional *R*-factors *R* are based on *F*, with *F* set to zero for negative *F*^2^. The threshold expression of *F*^2^ > σ(*F*^2^) is used only for calculating *R*-factors(gt) *etc*. and is not relevant to the choice of reflections for refinement. *R*-factors based on *F*^2^ are statistically about twice as large as those based on *F*, and *R*- factors based on ALL data will be even larger.

### Fractional atomic coordinates and isotropic or equivalent isotropic displacement parameters (Å^2^)

**Table:**

	*x*	*y*	*z*	*U*_iso_*/*U*_eq_	
O1	0.62487 (12)	0.84217 (13)	0.45326 (5)	0.0466 (3)	
O2	0.87018 (12)	0.68273 (16)	0.72482 (6)	0.0553 (3)	
C1	0.70272 (16)	0.66013 (17)	0.69096 (7)	0.0380 (3)	
C2	0.67929 (14)	0.72639 (15)	0.61301 (6)	0.0335 (3)	
H2A	0.7769	0.7829	0.5867	0.040*	
C3	0.51381 (14)	0.71094 (14)	0.57282 (6)	0.0307 (2)	
C4	0.49582 (15)	0.78093 (15)	0.48827 (7)	0.0336 (3)	
C5	0.31401 (17)	0.77041 (18)	0.44739 (7)	0.0418 (3)	
H5A	0.3094	0.6623	0.4125	0.050*	
H5B	0.2977	0.8763	0.4117	0.050*	
C6	0.15984 (16)	0.76306 (18)	0.50688 (8)	0.0447 (3)	
H6A	0.1523	0.8781	0.5366	0.054*	
H6B	0.0449	0.7447	0.4767	0.054*	
C7	0.18949 (16)	0.61092 (18)	0.56688 (8)	0.0440 (3)	
H7A	0.0917	0.6112	0.6070	0.053*	
H7B	0.1850	0.4952	0.5375	0.053*	
C8	0.36834 (15)	0.62853 (15)	0.61076 (7)	0.0355 (3)	
C9	0.39581 (17)	0.56299 (18)	0.68908 (8)	0.0449 (3)	
H9A	0.2986	0.5063	0.7157	0.054*	
C10	0.55946 (18)	0.57744 (19)	0.72955 (7)	0.0457 (3)	
H10A	0.5742	0.5315	0.7831	0.055*	
C11	0.9071 (2)	0.6037 (3)	0.80161 (9)	0.0775 (6)	
H11A	1.0326	0.6270	0.8176	0.116*	
H11B	0.8871	0.4741	0.7982	0.116*	
H11C	0.8271	0.6553	0.8422	0.116*	

### Atomic displacement parameters (Å^2^)

**Table:**

	*U*^11^	*U*^22^	*U*^33^	*U*^12^	*U*^13^	*U*^23^
O1	0.0447 (5)	0.0552 (6)	0.0401 (5)	−0.0057 (4)	0.0086 (4)	0.0067 (4)
O2	0.0402 (5)	0.0863 (8)	0.0392 (5)	0.0017 (5)	−0.0064 (4)	0.0004 (5)
C1	0.0357 (6)	0.0438 (6)	0.0344 (6)	0.0044 (5)	0.0005 (4)	−0.0048 (4)
C2	0.0312 (5)	0.0358 (6)	0.0338 (5)	−0.0018 (4)	0.0044 (4)	−0.0026 (4)
C3	0.0312 (5)	0.0288 (5)	0.0322 (5)	−0.0006 (4)	0.0033 (4)	−0.0018 (4)
C4	0.0368 (5)	0.0300 (5)	0.0341 (5)	0.0001 (4)	0.0032 (4)	−0.0031 (4)
C5	0.0452 (7)	0.0409 (6)	0.0390 (6)	−0.0030 (5)	−0.0065 (5)	−0.0005 (5)
C6	0.0332 (6)	0.0463 (7)	0.0542 (7)	−0.0003 (5)	−0.0063 (5)	−0.0041 (5)
C7	0.0320 (6)	0.0476 (7)	0.0524 (7)	−0.0083 (5)	0.0029 (5)	−0.0002 (5)
C8	0.0326 (6)	0.0342 (6)	0.0399 (6)	−0.0025 (4)	0.0052 (4)	−0.0010 (4)
C9	0.0423 (7)	0.0485 (7)	0.0442 (6)	−0.0052 (5)	0.0116 (5)	0.0080 (5)
C10	0.0506 (7)	0.0534 (8)	0.0332 (6)	0.0034 (6)	0.0060 (5)	0.0074 (5)
C11	0.0548 (9)	0.1375 (18)	0.0399 (7)	0.0233 (10)	−0.0073 (6)	0.0056 (9)

### Geometric parameters (Å, °)

**Table:**

O1—C4	1.2159 (14)	C6—C7	1.5175 (19)
O2—C1	1.3649 (14)	C6—H6A	0.9900
O2—C11	1.4159 (19)	C6—H6B	0.9900
C1—C2	1.3818 (16)	C7—C8	1.5070 (16)
C1—C10	1.3925 (18)	C7—H7A	0.9900
C2—C3	1.3907 (15)	C7—H7B	0.9900
C2—H2A	0.9500	C8—C9	1.3891 (17)
C3—C8	1.3994 (15)	C9—C10	1.3804 (19)
C3—C4	1.4887 (15)	C9—H9A	0.9500
C4—C5	1.5006 (16)	C10—H10A	0.9500
C5—C6	1.5198 (18)	C11—H11A	0.9800
C5—H5A	0.9900	C11—H11B	0.9800
C5—H5B	0.9900	C11—H11C	0.9800
			
C1—O2—C11	118.26 (12)	C5—C6—H6B	109.7
O2—C1—C2	115.69 (10)	H6A—C6—H6B	108.2
O2—C1—C10	124.58 (11)	C8—C7—C6	111.28 (10)
C2—C1—C10	119.73 (11)	C8—C7—H7A	109.4
C1—C2—C3	120.49 (10)	C6—C7—H7A	109.4
C1—C2—H2A	119.8	C8—C7—H7B	109.4
C3—C2—H2A	119.8	C6—C7—H7B	109.4
C2—C3—C8	120.53 (10)	H7A—C7—H7B	108.0
C2—C3—C4	118.63 (9)	C9—C8—C3	117.73 (11)
C8—C3—C4	120.83 (10)	C9—C8—C7	121.83 (10)
O1—C4—C3	120.99 (10)	C3—C8—C7	120.43 (10)
O1—C4—C5	121.25 (10)	C10—C9—C8	122.25 (11)
C3—C4—C5	117.75 (10)	C10—C9—H9A	118.9
C4—C5—C6	113.35 (10)	C8—C9—H9A	118.9
C4—C5—H5A	108.9	C9—C10—C1	119.27 (11)
C6—C5—H5A	108.9	C9—C10—H10A	120.4
C4—C5—H5B	108.9	C1—C10—H10A	120.4
C6—C5—H5B	108.9	O2—C11—H11A	109.5
H5A—C5—H5B	107.7	O2—C11—H11B	109.5
C7—C6—C5	110.04 (10)	H11A—C11—H11B	109.5
C7—C6—H6A	109.7	O2—C11—H11C	109.5
C5—C6—H6A	109.7	H11A—C11—H11C	109.5
C7—C6—H6B	109.7	H11B—C11—H11C	109.5
			
C11—O2—C1—C2	−174.47 (13)	C5—C6—C7—C8	56.14 (14)
C11—O2—C1—C10	5.8 (2)	C2—C3—C8—C9	−0.25 (17)
O2—C1—C2—C3	−179.84 (10)	C4—C3—C8—C9	178.68 (10)
C10—C1—C2—C3	−0.07 (18)	C2—C3—C8—C7	−179.62 (10)
C1—C2—C3—C8	0.18 (17)	C4—C3—C8—C7	−0.69 (16)
C1—C2—C3—C4	−178.76 (10)	C6—C7—C8—C9	150.91 (12)
C2—C3—C4—O1	3.71 (16)	C6—C7—C8—C3	−29.74 (16)
C8—C3—C4—O1	−175.24 (11)	C3—C8—C9—C10	0.20 (19)
C2—C3—C4—C5	−177.30 (10)	C7—C8—C9—C10	179.56 (12)
C8—C3—C4—C5	3.75 (15)	C8—C9—C10—C1	−0.1 (2)
O1—C4—C5—C6	−156.85 (12)	O2—C1—C10—C9	179.77 (12)
C3—C4—C5—C6	24.16 (15)	C2—C1—C10—C9	0.02 (19)
C4—C5—C6—C7	−53.97 (14)		

### Hydrogen-bond geometry (Å, °)

**Table:**

Cg1 is the centroid of the C1–C3,C8–C10 ring.

**Table:**

*D*—H···*A*	*D*—H	H···*A*	*D*···*A*	*D*—H···*A*
C11—H11C···O1^i^	0.98	2.38	3.3095 (18)	157
C5—H5A···Cg1^ii^	0.99	2.77	3.6730 (14)	152

Symmetry codes: (i) *x*, −*y*+3/2, *z*+1/2; (ii) −*x*+1, −*y*+1, −*z*+1.
